# Is AI a functional equivalent to expertise in organizations and decision-making? Opportunities and pitfalls for AI in the context of just transitions

**DOI:** 10.3389/frai.2025.1571698

**Published:** 2025-05-23

**Authors:** Marco Billi, Julio Labraña

**Affiliations:** ^1^Faculty of Agricultural Sciences, University of Chile, Santiago, Chile; ^2^Center for Climate and Resilience (CR2), University of Chile, Santiago, Chile; ^3^Transdisciplinary Systemic Research Hub (NEST.R3), Santiago, Chile; ^4^Faculty of Education and Humanities, University of Tarapacá, Arica, Chile

**Keywords:** intelligence, expertise, organizations, just transitions, complexity, science-policy, interface

## Abstract

The urgency of addressing climate change and achieving a just transition to sustainability has never been greater, as the world approaches critical environmental thresholds. While artificial intelligence (AI) presents both opportunities and challenges in this context, its role in organizational decision-making and expertise remains underexplored. This paper examines the interplay between AI and human expertise within organizations, focusing on how AI can complement or substitute traditional expertise across factual, temporal, and social dimensions. Drawing on Social Systems Theory, we argue that while AI excels in data processing and rapid decision-making, it falls short in contextual adaptation, long-term strategic thinking, and social legitimacy—areas where human expertise remains indispensable. And this is, we observe, particularly evident in problems connected with climate change and sustainability more broadly, where the tensions for organizational decision-making -and governance become even denser as much in the factual, temporal and social dimensions, making them into very complex, ‘super-wicked’, problem situations. Thus, there is a need to think more in detail about possible hybrid approaches, integrating AI’s computational strengths with human interpretive and adaptive capabilities, which may offer promising pathways for advancing organizational decision-making in the overly complex, wicked decision-making scenarios characteristic of just transitions. However, this requires careful consideration of power dynamics, trust-building, and the ethical implications of AI adoption. By moving beyond techno-optimism, this study highlights the need for a nuanced understanding of AI’s functional and social plausibility in organizational settings, offering insights for fostering equitable and sustainable transitions in an increasingly complex world.

## Introduction

With the world on the verge of surpassing the 1.5°C threshold set by the Paris Agreement and exceeding multiple planetary boundaries, the urgency of transitioning to sustainable development has never been greater. While past efforts have been insufficient, a profound transformation in production, consumption, and societal organization is imperative to achieve carbon neutrality and environmental sustainability.

Yet, sustainability is not merely about reducing emissions and pollution; it must also be just and inclusive. A just transition ensures that the burdens and benefits of change are equitably distributed, leaving no one behind. In this context, artificial intelligence (AI) emerges as both a potential catalyst and a challenge. On one hand, AI offers new efficiencies in production, energy management, and resource tracking, but on the other, its ecological footprint and disruptive effects on employment raise pressing concerns. AI itself is a driver of transition, particularly in reshaping labor and decision-making structures, making it crucial to examine how this shift can be made equitable.

The rapid evolution of AI—outpacing regulatory capacities—has fueled both optimism and anxiety. While some view it as a technological leap toward a better future, others warn of unregulated risks. The 2024 “Global Digital Compact,” established at the UN Summit for the Future in New York, represents an initial effort to harness AI’s potential while mitigating its threats in the pursuit of sustainability and equity.

However, meaningful action requires moving beyond hype to a deeper understanding of AI’s real impact on society and the conditions for a just transition. Much of the existing literature focuses on AI’s technical dimensions, often neglecting the broader socio-technical dynamics at play. Transformative shifts—particularly those that redefine production, consumption, and development paradigms—cannot be understood solely as technological processes. They are embedded within complex networks of science, regulation, industry, economics, and social expectations, unfolding through gradual, multi-scalar, and non-linear dynamics.^1^ In this sense, promoting a just transition—as well as tackling climate change and sustainability more generally- is at its core a matter of decision-making and governance (Agrawal et al., 2022; Underdal, 2010; Billi et al., 2021). And in modern society, a good part of decision-making and governance is made in, through or between organizations (Luhmann, 2018; Willke, 2006) so that understanding if and how AI development can impact—positively or negatively—organizational decision-making is very relevant for the research o just, sustainable and zero-carbon transitions.

This paper contributes to this discussion by examining the relationship between AI and expertise within organizations and reflecting on the implications—opportunities and challenges—it can bring to decision-making relating to climate change and sustainability. We argue that understanding expertise’s historical de-humanization within organizations is key to assessing AI’s role in a just transition. Using Social Systems Theory, we provide a sociological and historical perspective to counter the oversimplifications often present in AI debates, particularly the tendency to “over-humanize” both organizations and AI itself. Then, we look at how sustainability challenges may require rethinking the dichotomy between AI and human expertise, moving towards more ‘hybrid’ approaches and thus pushing forward the need of more research on how to design and implement effective and just forms of human-AI expertise hybridization.

The paper is structured as follows: Section II reviews dominant theories on technological singularity and AI’s impact on expertise within organizations. Section III draws on Social Systems Theory to contextualize the evolution of expertise and the pressures toward its de-humanization, while Section IV explores whether AI can functionally replace expertise in organizations, identifying its limits. With this theoretical background, Section V turns to the central question: what are AI’s opportunities and challenges in fostering a just transition to sustainability? Finally, Section VI offers concluding reflections and directions for future research.

## Artificial intelligence, expertise and organizational decision-making: a brief summary

The term *Artificial Intelligence* broadly encompasses various technologies, though most current applications revolve around machine learning—algorithms that refine performance through exposure to data without explicit programming. Since the 1950s, AI development has oscillated between phases of optimism (“AI springs”) and stagnation (“AI winters”), constrained by computing power, labor-intensive data preparation, and the brittleness of early systems (Schraagen and van Diggelen, 2021). A turning point arrived in the 2010s with big data and deep learning, which allowed neural networks to autonomously process vast datasets, reducing human intervention while introducing new challenges such as data dependence and opaque decision-making mechanisms (Jiang et al., 2022).

This progress has fueled a resurgence of speculation about AI’s long-term trajectory, including debates over superintelligence and technological singularity (Krüger, 2021). Perspectives vary widely: skeptics argue that AI’s advancement is overhyped and that true singularity remains a distant or unattainable goal, while proponents—including transhumanists—view it as an imminent and beneficial breakthrough. Meanwhile, critics warn of potential risks, ranging from job displacement to existential threats (Hoffmann, 2023). Although some foresee rapid progress, others highlight persistent limitations such as the finite availability of high-quality data and the growing computational costs of scaling AI models (Walsh, 2017).

AI’s role in decision-making has evolved in parallel. The first significant applications emerged in the 1980s with expert systems, which sought to encode human knowledge into structured AI models. These systems, however, proved limited in their application, leading to the refinement of knowledge-based systems and, later, the resurgence of AI-driven decision-making through deep learning (Duan et al., 2019). Despite these advances, concerns persist over AI’s capacity to replace human labor and the risks associated with autonomous decision-making, particularly in high-stakes areas such as healthcare, security, and governance (Pilling and Coulton, 2019).

In response, contemporary approaches increasingly emphasize hybrid models that integrate human expertise with AI capabilities. Many organizational decisions involve uncertainty, complexity, and ethical considerations, where AI’s analytical strengths can complement human intuition, experience, and contextual understanding (Trunk et al., 2020). This shift aligns with a broader redefinition of expertise, moving beyond static domain-specific knowledge to incorporate adaptive intelligence, intuitive reasoning, and interdisciplinary competencies (Carbonell and Dailey-Hebert, 2021).

Consequently, scholars and practitioners increasingly advocate for AI-human hybridization that acknowledges elements of singularity debates while preserving the unique strengths of human intelligence. As with past waves of automation, AI may not eliminate jobs outright but rather transform labor markets, reshaping the nature of expertise and the skills required for emerging roles (Jarrahi, 2018). While AI’s impact remains uncertain, its integration into organizational decision-making suggests a shift not toward full automation but toward redefining human labor and intelligence in an evolving technological landscape (Labraña and Bill, 2015).

## Organizations as social systems and the role of expertise

Niklas Luhmann’s Social Systems Theory offers a sociological framework for analyzing modern society as a system of communication (Luhmann, 2013). Rather than focusing on individuals or actions, this theory conceives society as constituted by communication. Within this framework, organizations are understood not as aggregates of persons or goals, but as specific types of social systems defined by their ability to produce decisions. From this perspective, organizations are forms of social systems that emerge to manage complexity and reduce uncertainty in modern, functionally differentiated societies. Unlike interaction systems or broader societal function systems—such as politics, economy, or education—organizations are problem-oriented systems that establish structured ways of coordinating communications through decisions. While organizations are not defined by a specific binary code, as function systems are, their operations depend on the continuous generation and stabilization of decisions, which in turn create their internal coherence against their environment (Luhmann, 2013). This approach has been extensively used to analyze the structural and operational logic of organizations, highlighting how decisions function as a mechanism of systemic closure and continuity (Andersen, 2003).

This focus on decision-making underlines the fundamental problem organizations face: the necessity of addressing and reducing overwhelming complexity while maintaining its coherence in a dynamic environment (Seidl and Becker, 2005). Decisions, as selective mechanisms, serve to filter possibilities by determining what aspects are included in communication and what is excluded. This ongoing process of selectivity underscores the fragility of organizational coherence, as every decision, by simplifying complexity, simultaneously excludes alternatives, thereby generating risks that in turn demand further decisions in a self-producing cycle of further decisions. In this sense, organizations are not stable entities, but dynamic systems whose continuity depends on their capacity to recursively produce decisions (Nassehi, 2005; Seidl and Mormann, 2014; Luhmann, 2020).

Expertise must be understood within this broader context as a phenomenon that does not represent an inherent feature of organizations or their initial development. In pre-modern societies, coordination within pre-organizational forms—such as guilds, religious orders, or early bureaucracies—relied heavily on tradition, charisma, or personal authority, which tied decision-making and knowledge systems to individual actors and culturally embedded norms (Weber, 1978). However, as societal complexity increased, these mechanisms proved insufficient to address the demands of more differentiated and dynamic environments. Expertise emerged as an institutionalized resource in early modernity, serving as a response to this growing challenge, decoupling decision-making from individual authority and anchoring it in specialized systems of knowledge (Meyer and Rowan, 1977). This shift not only allowed organizations to manage complexity more effectively, in a way less context-dependent, but also contributed to the de-humanization of organizational dynamics, as the reliance on personal relationships and intuitive authority was replaced by impersonal, procedural, and often automated frameworks of knowledge production and decision-making (Warner, 2007). Expertise thus became embedded within roles, credentials, and institutional structures, transforming organizations into systems increasingly oriented towards predictability, while subordinating interpersonal or traditional forms of coordination to the authority of specialized knowledge systems that claimed a better understanding of their respective environments (Collins, 1979).

Functional differentiation—the process by which society becomes segmented into autonomous subsystems, each with its own rationality, language and rules, such as law, economy, education, and science (Luhmann, 1982)—has been pivotal in shaping the relationship between expertise and the emergence of modern organizations. As each subsystem developed its own distinct operational logic, organizations emerged as mediating structures tasked with interpreting and implementing these logics in context-specific ways (Labraña et al., 2025). Financial institutions, for example, became critical to the economy by operationalizing financial transactions and managing economic flows, while schools aligned themselves with the education system by translating pedagogical theories into structured learning practices, and courts embedded within the legal system transformed legal norms into decisions on concrete cases. In each of these instances, organizations required specialized expertise to bridge the gap between the abstract, often self-referential operations of societal subsystems and the concrete, practical demands of their environments. Expertise thus became indispensable, enabling individuals within organizations to fulfill their expected roles while allowing organizations to adapt and coordinate in response to the increasingly abstract and complex demands arising from the expansion of functionally differentiated systems (Zald and Lounsbury, 2010; Labraña and Vanderstraeten, 2020).

Expertise thus became the primary mechanism through which organizations structured their relationships with the broader societal systems they were embedded in (Luhmann, 2013). By doing so, expertise enables organizations to achieve operational stability by systematically reducing complexity across the three key dimensions of meaning: factual, temporal, and social. In the factual dimension, expertise allows organizations to presuppose a stable and predictable reality by providing specialized knowledge that delineates domains of relevance, framing problems and solutions within bounded contexts. This stabilization of communication reduces the need for continuous renegotiation of facts, creating a foundation for shared understandings among organizational members (Simon, 1991; Weick, 1995). For instance, in engineering firms, expertise defines technical parameters, enabling clear problem identification and reliable solutions (Bucciarelli, 1994). Similarly, in medical organizations, expertise grounds diagnoses and treatments in evidence-based practices, fostering a common understanding of health and disease that shapes operational decisions (Berg, 1997). Lastly, in schools, expertise establishes pedagogical frameworks that stabilize teaching methodologies, fostering shared educational goals among educators and students (Shulman, 1987). Through these mechanisms, expertise aligns organizational practices with the complex demands of the societal systems they are embedded in, ensuring that responses are not only legitimate but also help reduce environmental complexity in ways that are both effective and socially convincing.

In the temporal dimension, expertise operates as a dynamic and continuously evolving resource for organizational decisions, distinguishing itself from forms of knowledge that often claim timeless validity. Its relevance lies in its ability to adapt to changing circumstances, functioning as a self-substitutive order that perpetually renews itself through the ongoing refinement of the theories and methodologies upon which it is ultimately based (Luhmann, 1990). For instance, legal expertise evolves to integrate new regulations and precedents, while technological expertise advances alongside innovations in tools and systems to retain its social effectiveness (Teubner, 1987). Central to this process is professional training within educational institutions, which serves as the primary mechanism for the continual updating and refinement of expertise. Schools and universities, especially, play a crucial role by establishing standardized frameworks and methodologies designed to equip individuals with the knowledge needed to operate as experts in their respective fields, ensuring that expertise remains a relevant, adaptive, and useful resource in complex organizational environments (Brown, 2001).

In the social dimension, expertise legitimizes decision-making processes within organizations by establishing hierarchies of knowledge and authority, where the ability to decide is not solely based on possessing specialized knowledge but also on being recognized as having the authority to do so (Luhmann, 2000). This recognition functions as a legitimizing mechanism that is not merely an objective reflection of competence but also a socially constructed attribution of authority (Stichweh, 1994; Eyal, 2019). In this sense, legitimacy is not derived from expertise alone but from the institutional and communicative processes that attribute trustworthiness and decision rights to certain roles or individuals. In turn, this recognition creates distinctions between experts and non-experts, facilitating the coordination of decisions and reducing complexity within organizations. Based upon this, expertise fosters trust and accountability by enabling the delegation of responsibilities and the implementation of decisions within a framework of legitimacy, reinforcing organizational coherence and ensuring the effective allocation of tasks and resources toward shared objectives (Bunz, 2014). For example, in hospitals, the expertise of doctors and nurses—validated through certification and training—ensures that medical decisions are both credible and authoritative, maintaining trust among organizational members and external stakeholders (Freidson, 1970). Likewise, in educational institutions, the expertise of teachers and administrators—validated through formal qualifications and professional development—provides a foundation for decision-making processes that guide curriculum design, student assessment, and resource allocation (Hoyle and Wallace, 2005). By clearly defining roles and responsibilities based on expertise, organizations reduce uncertainty, minimize conflicts over who has authority to decide on which topics, and establish a framework for achieving their goals, reinforcing their capacity to respond to internal and external changes.

## Artificial intelligence as a (partial) functional equivalent of expertise in organizational decision-making

The increasing adoption of AI in organizational settings has prompted debates about whether it can serve as a functional equivalent to human expertise. As explored in the previous section, expertise has historically emerged as a mechanism to reduce complexity in organizations, addressing uncertainty through the factual, temporal, and social dimensions. AI, with its capacity for data analysis, pattern recognition, and automation, appears to replicate certain functions of expertise. However, when examined in light of a sociologically-grounded understanding of expertise as outlined earlier, AI reveals limitations that challenge its ability to serve as an equally comprehensive substitute.^2^

In the factual dimension, human expertise combines generalization and specificity to address organizational challenges within bounded contexts. This capacity for contextual adaptation allows experts to frame problems in ways that are both precise and actionable, drawing on abstract principles and practical experience. By contrast, AI systems focus on generalizable patterns derived from vast datasets (LeCun et al., 2015). As already discussed above, in the first eras of AI, this training often made these systems overfitted to specific problem-situation, completely losing any ability to translate knowledge from one domain to the other (i.e., they only had a very restricted domain expertise, with no general expertise). This was called ‘brittleness’. While contemporary approaches to AI, and particularly deep learning, have overcome some of these limitations thanks to the use of a much broader base of data and parameters, they fundamentally still rely on the learning of specific ‘rules’ and patterns, as opposed to what human experts do by assigning a ‘meaning’ to data which can actively connect one domain of knowledge and learning with others through higher-level cognitive architectures, that these systems lack. The deep learning approach thus excels in identifying trends or optimizing routine processes, but it often fails to account for the specificities that arise in complex or novel situations. For example, a financial algorithm may efficiently detect fraudulent transactions by analyzing patterns across thousands of data points but may struggle to account for contextual nuances, such as the socio-economic conditions influencing certain behaviors (O'Neil, 2016). Similarly, in the healthcare sector, AI tools may accurately flag anomalies in diagnostic imaging; however, they often fail to integrate this information with patient histories, physician observations, or the socio-cultural contexts that influence care—unless explicitly trained to do so (Obermeyer and Emanuel, 2016). Even more relevant, in the field of artistic creation, AI demonstrates the ability to generate texts that give the impression of creativity. However, these outputs often lack the deeper contextual awareness and intentionality that has historically defined proper human artistic expression.

This emphasis on generalization limits AI’s ability to generate the context-sensitive relevance required for effective organizational decision-making. Expertise, in contrast, goes beyond merely providing answers; it involves identifying the limitations of existing knowledge and bridging these gaps through experiential insights. AI’s reliance on large-scale datasets creates a dependency fundamentally distinct from the contingency-responsive and adaptive qualities inherent in human expertise (Stinson and Vlaad, 2024). As discussed in Section III, expertise reduces complexity in organizational operations by presupposing a relatively stable world and integrating theoretical knowledge with practical experience to frame and address relevant issues. AI, however, lacks such foundational presuppositions, making it highly susceptible to incomplete, biased, or poorly contextualized data —a vulnerability that has garnered growing attention (Zou and Schiebinger, 2018). As a result, the insights generated by AI risk being not only irrelevant but also potentially counterproductive to organizational decision-making anytime the decision involves this kind of context-specificity, or higher degrees of general expertise as compared to domain expertise, undermining its capacity to address context-specific challenges and ensure the relevance and effectiveness of its actions.

Furthermore, AI’s reliance on external inputs highlights its inability to autonomously delineate and prioritize relevance within complex organizational environments. This dependency renders AI incapable of independently addressing ambiguity or adapting to contexts where information is incomplete, conflicting, or fluid, as it is increasingly evident in organizational decision-making (Kahneman and Klein, 2009). Unlike human expertise, which leverages experiential insights and reflection to discern relevance and establish priorities, AI systems are entirely constrained by the quality, scope, and structure of the data they are provided. This reliance not only limits their capacity to make judgments but also prevents them from accounting for variables that lie outside predefined parameters, reducing their effectiveness in new and unpredictable scenarios. Similarly, it also makes them strongly subject to underlying biases in the data, something very visible in the different forms of ‘automated discrimination’ that AIs inherit from their data (Heinrichs, 2022).

In the temporal dimension, AI clearly surpasses human expertise any time a very quick decision needs to be made considering a large amount of new information, that humans would not be able to process. But in organizations, expertise is not only a mechanism to make quick decisions; rather, and much more importantly, it serves to reduce complexity by fostering trust in human judgment, particularly in uncertain contexts. Unlike AI, which operates within predefined parameters, human expertise is inherently dynamic and adaptive, drawing on interpretive processes that integrate past experiences with plausible anticipations of the future. This ability to contextualize decisions temporally enables expertise to address immediate challenges while considering their broader implications for future scenarios. By aligning present actions with long-term objectives and strategies, expertise equips organizations to confront uncertainty with confidence, ensuring that decisions are guided by both historical insights and forward-looking perspectives. In contrast, AI operates through a logic of sufficiency rather than interpretive anticipation. While machine learning systems can adapt by incorporating new data, this process is fundamentally reactive, relying on existing patterns and inputs. As a result, AI lacks the critical proactive capacity to assess emerging or unforeseen conditions (Dreyfus and Dreyfus, 2005).

Equally important, trust in expertise is deeply rooted in its capacity to justify decisions and respond effectively to unanticipated developments. Experts do not merely predict outcomes; they provide explanations that frame uncertainty in meaningful ways, fostering confidence and enabling contingency planning. In contrast, AI systems, while capable of producing statistically robust outputs, often lack the interpretive depth necessary to contextualize their recommendations. The opacity of many algorithms—the so-called “black box” problem (Bathaee, 2018)—further erodes trust by concealing the reasoning behind their conclusions. This lack of transparency poses significant challenges for organizations, particularly in high-stakes contexts where accountability, adaptability, and a clear rationale for decisions are critical. Without the ability to articulate why a specific course of action is recommended, AI systems risk being perceived as unreliable, limiting their utility in contexts requiring rather explicit interpretive insights (Ananny and Crawford, 2018). In this sense, AI systems are somewhat more similar to ‘intuitive’ expertise, or ‘gut feeling’, which while broadly used in decision-making (and arguably, one of the most significant components of human expertise) also shares this lack of clear explain ability. However, even intuitive expertise can ultimately be explained, understood and even predicted (and abundantly subject to measurement and testing, see Section 2) based on identifiable sets of human characteristics, which makes it possible to anticipate that some ‘person’ will be likely more expert than another in certain tasks, as well as to foster and nurture expertise, both in the education system and within organizations. This is not the case with IA: while AI ‘learns’, and AIs with more parameters or more data allegedly learn more and faster, there are still not clearly defined attributes that can help an observer know beforehand which AI will be more expert at what, and even, whether all times the same AI will be called -each of this is, in some way, a new individual ‘expert’ that learns from the specific interaction but cannot be replicated in future interactions- it will always show the same expertise. Steps are being done in this direction, and prompt engineering’ may somewhat solve this, but still strongly relying on human intervention.

Additionally, the institutional trust-building mechanisms underpinning human expertise is fundamentally absent in AI systems. Expertise is deeply embedded within professional networks, credentialing processes, and institutional frameworks that collectively establish its legitimacy and ensure its accountability (Brint, 1994). These structures not only validate and update expert knowledge but also create mechanisms for holding experts responsible for their decisions, thereby fostering confidence in their guidance. AI, by contrast, functions as a technical artifact, disconnected from these institutional connections, which makes it significantly more challenging to perceive its outputs as a reliable foundation for long-term decision-making. While AI excels at optimizing specific tasks within well-defined parameters under quick-answer problem situations, its inability to participate in the broader dynamics of social trust highlights a limitation in its capacity to replace human expertise in longer-term contexts that require a broader picture (Pasquale, 2015).

In the social dimension, expertise serves not only as a repository of specialized knowledge but also as a legitimizing mechanism within organizational hierarchies. It gains recognition and validation through the distinction between experts and non-experts, creating a structured framework for trust, authority, and accountability. This distinction is essential for organizational operations, as it facilitates the delegation of decision-making and the establishment of clear lines of responsibility. AI, however, disrupts this social framework. As a non-human system, it lacks the relational and institutional positioning that underpins human expertise, making it incapable of occupying the role of an “expert” in the traditional sense. While advanced AI systems such as ChatGPT can simulate dialogue, offer justifications, and respond to challenges to some extent, these interactions remain only partially embedded in the social and institutional contexts necessary for conferring legitimacy. As noted, legitimacy arises not merely from functional outputs but from the social attribution of trust, responsibility, and accountability—dimensions that AI is not capable of fulfilling autonomously. It therefore continues to function as a tool whose outputs require human interpretation and mediation (Binns, 2018).

A key issue in this regard is the indeterminacy of AI’s “unmarked side.” Expertise relies on clearly defined boundaries between what is known and what remains unknown, along with the ability to articulate those boundaries transparently. Human experts do not simply provide answers; they also inevitably communicate the limitations of their knowledge, making the scope and constraints of their expertise explicit. In contrast, AI operates without such transparency. The already mentioned “black box” nature of many AI systems obscures the assumptions underlying their outputs and makes it difficult to identify the limits of their knowledge. This opacity disrupts the traditional distinction between experts and laypersons, creating uncertainty about AI’s appropriate role within organizational hierarchies and how its outputs should be evaluated (Ananny and Crawford, 2018). That is: AI is both an extremely knowledgeable specialist and a stupid advisor.

Moreover, the social dynamics of expertise involve more than the validation of knowledge—they also encompass the coordination of diverse perspectives within organizations. Human experts play a critical role as mediators, integrating insights from various domains to facilitate collaboration, alignment, and consensus-building. They do so not only by ‘knowing’ (and being expert) at all the domains, but even more importantly, engaging in team work, creative collaboration and knowledge sharing with other areas. In contrast, AI systems lack this capacity. While they can generate highly individualized information, AI systems do not engage in the processes that harmonize knowledge with organizational objectives or resolve conflicting perspectives, limiting their effectiveness in multi-stakeholder environments and resulting in less legitimate outcomes (Jarrahi, 2018).

## Organizational decision-making in the face of sustainability and climate change: the promise of AI

Having understood to what extent and with which caveats can AI complement or integrate with traditional human expertise in organizational decision-making, we now turn to the central question of the manuscript: *what challenges and opportunities does this imply for sustainability and climate change?* In particular, how—to which degree and in which direction—the expansion and potential hybridization of expertise may have an effect on the (organizational) decision-making dilemmas related to the attempt to steer and accelerate sustainable transitions in our societal, technological and ecological environments? In previous works (Billi et al., 2020;^9004,9003^), we have performed a deep reflection on these dilemmas, using an analytical framework very similar to the one we have discussed so far. In these reflections, we have employed the term ‘governance’ to refer to the whole array of decision-making processes related to sustainable transitions, including both decisions that are taken in the domain of traditional for-profit and non-for-profit organizations, in the public arena (by State and public organizations, as well as political institutions) and in the different emerging realms of network-like quasi-organizations that often populate the field of sustainability. This implies broadening the scope of analysis to a broader meaning of organization and decision-making, which however can learn a lot from all that has been studied in terms of expertise, and its relationship with AI, in the narrower setting of conventional organizations.

In these studies, we have argued that decision-making related to sustainability transitions and climate change mitigation or adaptation, and thus expertise related to said decisions, is fundamentally faced with three dilemmas, each of which implies a specific ‘tension’ that decisions and expertise need to navigate, related to the same three dimensions discussed above: factually, in terms of the tension between the universality and specificity of the problem and knowledge on which decisions need to be made; temporally, the tension between long-term and short-term horizons of decision, and related to this, between the continuity of drive between decisions taken at different times and the need to adjust to changing circumstances; and socially, the tension between the coordination of decisions taken by different actors, and thus, also the possibility of some actors of restricting or steering decisions of others, and the need to maintain a degree of agency and autonomy of each individual decision maker (and thus, take advantage of their specific expertise).

In particular, our claim was that the quest for sustainability transitions applies an increasing pressure on both sides of the spectrum of each of these decision-making tensions, and thus the problem of governance (but also of expertise) becomes how to balance between them in these growingly complex conditions. This is, for many, one of the core issues that requires facing in order to face problems related to climate change -and sustainability more broadly: linear, structured, problem-solving thinking is not enough to fathom -let alone solve them. In fact, it can often lead to worsening them or creating new ones (Gupta, 2016; Lazarus, 2008; Voss et al., 2006). And it is also why, while the COVID-19 pandemics, despite its tragedy and impact, could be mostly ‘solved’ in less than 2 years, while climate change has still no clear ‘solution’ in sight despite knowledge of it having been around for more than a century, and counting (Billi et al., 2024b).

In the factual dimension, decisions regarding just transition oriented to sustainability and climate change require specificity because they relate to multiple and different domains, systems, scales, each implying its own kind of expertise. For instance, a transition in the ‘energy system’ requires to consider economical, technical, ecological, socio-cultural, legal and political factors, as they accrue as much at the global level, as at the national and subnational ones (Klein, 2020; Saruchera, 2025). No single set of decisions will be the best one to push forward transitions across all these contexts, different variables and knowledges need to be balanced, and this deeply challenges the cognitive limitations of human experts, which tend to have a limited grasp of the knowledge required in each of these domains, and are likely expert at most in a subset of them.

However, at the same time, these decisions need also to be able to transcend their contexts, because of the high interdependence of actions taken in each domain and scale: impacts on one sector can generate chain effects on others; measures that respond to current challenges at some scale could generate counterproductive consequences in other scales and actions that are appropriate for a certain group or sector may be negative for others. Even improved as it is, AI remains too brittle to be able to deeply tackle these interdependencies, and it lacks access to a meaning-making mechanism that can allow it to interpret and understand how these different decisions may interact with each other in different contexts. However, it can provide a vast access to data and knowledge which can help human experts to make sense of this complexity. Here, a hybridization of human (both intuitive and rational) general and context-sensitive expertise and artificial domain-specific expertise could be beneficial in that it may be able to expand the cognitive span of decision-making systems beyond the traditional limitations and thus capture as much domain knowledge as needed while also retaining the ability to read between domains, much similar to the hope that was once upon a time invested in the development of ‘expert systems’. However, for that to happen, the human expert should remain in charge and at the drive, resisting the temptation of taking for granted patterns and suggestions made by AI systems, and instead guiding the search for new and more reflexive ways of understanding the complexity and making connections. In this framework, AI should primarily serve as a tool and an assistant to human expertise, augmenting rather than replacing the interpretive strengths of human decision-makers.

In the temporal dimension, decisions regarding just transitions imply a high degree of anticipation, long-term perspective and tolerance to uncertainty. Not only sustainability and climate change imply slow-moving variables, so that their causes and effects require to take into account decades- and often centuries-long timeframes. But also, transitions required to tackle them may require decades to happen, needs to nidify strategies into strategies and anticipate future scenarios which are unclear in their probability and even in the assumptions that are made to create them (sometimes referred to as ‘deep uncertainty’ Haas et al., 2023). Even more crucially, transitions are ill-structured problem situations, or “wicked problems” as they tend to often be called (Termeer et al., 2015) -or even “super-wicked,” in the case of climate change (Gilligan and Vandenbergh, 2020). AI is not well equipped to deal with these kinds of problems, and truth be said, not all humans are. In fact, it is often implied that these problems require reframing our way of thinking, deepening our critical reflexivity, inter and transdisciplinary attitude and advancing new form of collaboration and leadership (Earle and Leyva-de la Hiz, 2021). Expertise, particularly adaptive expertise, must then be nurtured to face these problems, requiring not only human decisions, but decisions that are trained and sensitivities to open up to these new forms of thinking. But at the same time, just transitions also require short-term decisions, and in fact, it requires to quicken and multiply decision-making power to be able to adjust almost in real time to changing scenarios and conditions, in a way and pace which humans cannot readily adopt. For instance, optimizing energy efficiency, or water use, or organizing circular economy structures and so on, requires very fast and broad-spanning decisions on multiple contexts and places at once. This does not necessarily require long-term thinking, but rather rapid data processing and memory, qualities in which AI systems excel (Haider et al., 2024; Zejjari and Benhayoun, 2024). So in the temporal dimension, hybridization should take at the same time the role of human expertise enhancement through AI, providing scenarios, data exploration and management tools to foster future-thinking, and replacing of humans by AI in routinary, quick-thinking tasks but with the possibility of overriding these when intuitive expertise tells otherwise.

Finally, in the social dimension, sustainability and climate change problems face not only a multiplicity of decision-makers, as they often require actions to be taken in a coherent and collaborative manners between public institutions, private enterprises, community members and so on, but also inherent and sometimes unsurpassable trade-offs, ‘hard choices’, contrasting values and worldviews, and no-size-fits-it-all solutions, that make all decision-making situation in this context inherently controversial and open-ended (O'Brien et al., 2009; Sapiains et al., 2020). Thus, the problem is how to include multiple perspectives, so that decisions not only make sense but also ensure their legitimacy and ownership by these different groups, while at the same time allowing that actors are able to coordinate and act in a timely and relatively orderly manner, in the face of joint problems and (limited) common resources.

In this context, AI is not up to the task, not alone at least. Replacing human decisions for AI systems may seem an attractive way out to some, removing the alleged ‘bias’ of human decisions to specific factions or worldviews, but what it ultimately does, is promoting a cold, context- and socially-insensitive form of technocracy. As discussed above, while AI does exude some sense of authority or legitimacy because of its perceived ‘objectivity’, this does not apply in overtly conflicted situations in which attention to subjectivity and controversies is fundamental for decisions to be considered legitimate. Moreover, as also discussed above, excessive trust on the objectivity of AI may also be misguided, as AI systems ultimately take in the inputs that they receive and derive patterns from them, without any ability to identify potential biases or discriminations that these may hide (either unintentionally or deliberately). On the other hand, AI systems can have a role here in expanding the accessibility of knowledge and expertise. As also discussed in the factual dimension, in complex problem-situations, not everybody can have access to all the knowledge needed to make a decision, and particularly, most people will probably have no training on most of the technical aspects of a decision, making human-only approach prone either to technocratic exclusion, or to populist rhetoric, e.g., oversimplifying myths and post-truths. In fact, even after decades of scientific and political work over this, many people still do not get a deep understanding of sustainability and climate change processes, and climate skepticism remains rampant (Dunlap, 2013). AI can here help by translating and making rapidly accessible deeper forms of knowledge to people that go beyond their individual sphere of expertise, so they can engage in more productive and informed dialogue and deliberation with their peers. However, this would require incorporating more explicitly training in use of AI -and also, in critical appraisal of AI ‘truths’ into both higher education and adult specialization curricula, which would also help in shifting capacities required to support inclusive and just transition processes.

## Conclusion

This paper examined the opportunities and challenges of AI in shaping a just transition to sustainability, particularly regarding its role as a partial alternative to human expertise within organizations. We have argued that expertise functions as a key mechanism for reducing complexity in decision-making, defining problems and solutions, adapting to change, and legitimizing decisions. AI, while useful in processing data, identifying patterns, and facilitating accessibility, cannot fully replace human expertise due to technical and social plausibility limitations. Effective AI integration requires developing new forms of collaboration between AI and human decision-makers—ranging from assistance to hybridization and supervised substitution—while simultaneously advancing human expertise to address the growing complexities of the world and support just transitions.

As discussed in the previous section, hybridization is required to respond to the growing complexity, rapidity, uncertainty and policontextuality of decision-making challenges, which becomes even more relevant in the frame of super-wicked problems such as climate change and other sustainability issues. Combining human and IA expertise would bring in this case not only a way of fostering the compatibility between human and AI expertise in organization, but also ways to harness this in the context of the green transition and adaptation strategies required by climate change and other sustainability issues.

However, as already noted, hybridization between human and artificial intelligence can take multiple forms—ranging from context-dependent procedures such as the interactive division of tasks, to AI-enhanced access to information, delegation of routine responsibilities, and more integrated workflows that enable the co-construction of knowledge and joint task execution. These models vary in their effectiveness and feasibility across different settings, highlighting the need for further research into the specific forms of hybridization most conducive to promoting just and sustainable transitions. Crucially, all such approaches require a rethinking of how current and future workforces are trained. This is particularly pressing in the context of green transitions, where occupational reorientation toward climate-compatible roles is rapidly becoming a central challenge. While our analysis highlights the limitations of AI in replicating the social and interpretive dimensions of human expertise, we also acknowledge that in certain well-structured, high-volume decision environments, AI systems may achieve a degree of autonomy or functional legitimacy—especially when supported by robust validation procedures, transparency protocols, and effective human oversight. Future research should critically investigate these scenarios to understand the institutional, technical, and social conditions under which AI might reliably assume roles traditionally reserved for human experts, without compromising trust, accountability, or ethical integrity.

Similarly, future research should explore how different organizations incorporate AI to advance just transitions, particularly in human-centric fields like education and healthcare, where ethical judgment and empathy remain irreplaceable. Another critical issue is trust—AI adoption depends not only on technical proficiency but also on its perceived legitimacy. Skepticism persists, warranting further study on whether it stems from AI’s limitations, its perceived inferiority to human expertise, or broader societal concerns. Additionally, the power dynamics of AI implementation must be further examined, as AI can either reinforce hierarchical structures or democratize access to expertise, impacting equity and justice in sustainability transitions.

The discourse on AI is often steeped in grand expectations or dramatic concerns, where lofty aspirations and dystopian fears outpace reality. Organizations stand at the crossroads of these ambitions, translating ideals into practice of day-to-day work and workforce management. In this context, however, insufficient attention has been put so far on the role, opportunities and challenges that the incorporation of AI-assisted decision and the hybridization of human and AI expertise can have on fostering more grounded and informed decisions in the context of complex, (super-)wicked problems such as climate change and sustainability. This study moves beyond promises, anchoring the conversation in functionality and plausibility—what AI can truly offer, rather than what it merely envisions. In this pursuit, innovation alone is not enough; a deeper understanding of the social, cultural, and political landscapes in which AI unfolds is essential. Only by acknowledging these complexities can AI’s role in sustainability and climate change transcend rhetoric and become a force for meaningful transformation.

To advance in this direction, it is essential to foster interdisciplinary collaboration among computer scientists, organizational theorists, and sustainability scholars to develop context-sensitive frameworks for human–AI interaction. Practical experimentation through pilot initiatives—particularly in sectors such as urban planning, renewable energy, and climate governance—holds particular promise and can yield valuable insights into how hybrid systems function in real-world decision-making environments. In parallel, policy-oriented research should examine the regulatory, institutional, and normative infrastructures needed to ensure that AI implementation is consistent with democratic values, social inclusion, and environmental priorities. Addressing these challenges requires more than technical innovation; it demands a fundamental transformation in professional cultures, organizational learning, higher education, and accountability frameworks. Only through such integrated and reflexive efforts can AI serve as a meaningful contributor to just, sustainable and climate-neutral transitions.

## Footnotes

^1^Admittedly, these kind of considerations have a much broader application than sustainability or climate change issues. Readers may find these arguments interesting also for other topics of research. However, in this paper we decided to focus on this particular framing as questions of IA and expertise in organization and decision-making tend not to be sufficiently considered in sustainability and climate change literature, and we believe our approach may provide useful insights for this field, as is discussed at length below.

^2^Of course, this ‘equivalence’ between AI and human expertise is only partial, and contingent to specific contexts (e.g., specific topics or functions, ‘tactical’ instead than strategic decisions, ‘hard’ instead than ‘soft’ skills and so on). That is in part what the discussion between ‘specific’ AI and ‘general’ AI (AGI) (Emmert-Streib, 2024): the long-waited—or feared—promise of AGI is that it can substitute human expertise across the whole spectrum, and flexibly through different fields or decision-making situations. But all forms of AI, from search-aid chat-bots to ‘expert systems’ to enhanced reality to autonomous driving- are in some way a form of substituting ‘some’ kind of expertise in ‘some’ decision-making situation, and one of the main objectives of AI development has been indeed to expand the scope and reduce the ‘brittleness’ (that is, the lack of flexibility and generalizability) of AI in ever-more complex and broader decision-making situations.
